# Effectiveness of the XBB.1.5 COVID‐19 Vaccines Against SARS‐CoV‐2 Hospitalisation Among Adults Aged ≥ 65 Years During the BA.2.86/JN.1 Predominant Period, VEBIS Hospital Study, Europe, November 2023 to May 2024

**DOI:** 10.1111/irv.70081

**Published:** 2025-03-09

**Authors:** Liliana Antunes, Madelyn Rojas‐Castro, Marcos Lozano, Iván Martínez‐Baz, Isabel Leroux‐Roels, Maria‐Louise Borg, Beatrix Oroszi, Margaret Fitzgerald, Ralf Dürrwald, Ligita Jancoriene, Ausenda Machado, Goranka Petrović, Mihaela Lazar, Lenka Součková, Sabrina Bacci, Jennifer Howard, Nuno Verdasca, Luca Basile, Jesús Castilla, Silke Ternest, Aušra Džiugytė, Gergő Túri, Roisin Duffy, Carolin Hackmann, Monika Kuliese, Verónica Gomez, Zvjezdana Lovrić Makarić, Alexandru Marin, Petr Husa, Nathalie Nicolay, Angela M. C. Rose, VEBIS SARI VE network team, Itziar Casado, Aitziber Echeverría, Camino Trobajo‐Sanmartín, Manuel García Cenoz, Guillermo Ezpeleta, Carmen Ezpeleta, Miguel Fernández‐Huerta, Ana Navascués, Evelyn Petit, Marijke Reynders, Melanie Delvallee, Pierre Struyven, Charlotte Martin, Nicolas Dauby, Yama Toure, Helio Correia Cesar, Coca Necsoi, Leslie Andry, Benedicte Delaere, Marc Bourgeois, Benédicte Lissoir, Catherine Sion, Xavier Holemans, Reinout Naesens, Eva Bernaert, Door Jouck, Koen Magerman, Marieke Bleyen, Marlies Blommen, Laurane De Mot, Nathalie Bossuyt, Sarah Denayer, Yinthe Dockx, François Dufrasne, Anna Parys, Sébastien Fierens, Claire Brugerolles, Hilde Jansens, Sien De Koster, Veerle Matheeussen, Thomas Demuyser, Arne Witdouck, Els Van Nedervelde, Lucie Seyler, Siel Daelemans, Svea Geeroms, Arne Vilain, Isabel Leroux‐Roels, Pascal De Waegemaeker, Silke Ternest, Tanya Melillo, John‐Paul Cauchi, Stephen Abela, Gerd Xuereb, Judit Krisztina Horváth, Katalin Kristóf, Bánk Fenyves, Csaba Varga, Krisztina Mucsányiné Juhász, Katalin Krisztalovics, Terra Fatukasi, Lisa Domegan, Joan O’Donnell, Silke Buda, Kristin Tolksdorf, Ute Preuss, Djin‐Ye Oh, Janine Reiche, Torsten Bauer, David Krieger, Fausta Majauskaite, Ieva Kubiliute, Birute Zablockiene, Rolandas Zablockis, Goda Slekyte, Giedre Cincileviciute, Aukse Mickiene, Roberta Vaikutyte, Ana Paula Rodrigues, Débora Pereira, Margarida Tavares, Susana Costa Maia e Silva, Paula Pinto, Cristina Bárbara, António Pais de Lacerda, Raquel Guiomar, Camila Henriques, Nuno Verdasca, Ivan Mlinarić, Iva Pem Novosel, Irena Tabain, Diana Nonković, Petra Tomaš Petrić, Josipa Radas, Ivana Bočina, Svjetlana Karabuva, Mihaela Čikeš, Suzana Mladinov, Matea Nikolić, Ana Brnas, Antonija Medić, Joško Markić, Ivana Jukić, Ina Tomas, Marija Tonkić, Corneliu Popescu, Grațiela Târdei, Alma Gabriela Kosa‐Tudor, Simin Aysel Florescu, Emanoil Ceausu, Isabela Ioana Loghin, Mihaela Catalina Luca, Carmen Mihaela Dorobăț, Sorin Dinu, Catalina Pascu, Alina Ivanciuc, Iulia Bistriceanu, Mihaela Oprea, Maria Elena Mihai

**Affiliations:** ^1^ Epiconcept Paris France; ^2^ National Centre for Epidemiology Institute of Health Carlos III Madrid Spain; ^3^ Consortium for Biomedical Research in Epidemiology and Public Health (CIBERESP) Madrid Spain; ^4^ Instituto de Salud Pública de Navarra – IdiSNA Pamplona Spain; ^5^ Department of Infection Control Ghent University Hospital Ghent Belgium; ^6^ Center for Vaccinology Ghent University and Ghent University Hospital Ghent Belgium; ^7^ Infectious Disease Prevention and Control Unit (IDCU) Health Promotion and Disease Prevention Msida Malta; ^8^ National Laboratory for Health Security, Epidemiology and Surveillance Centre Semmelweis University Budapest Hungary; ^9^ Health Service Executive‐Health Protection Surveillance Centre (HPSC) Dublin Ireland; ^10^ National Reference Centre for Influenza Robert Koch Institute Berlin Germany; ^11^ Clinic of Infectious Diseases and Dermatovenerology, Institute of Clinical Medicine, Medical Faculty Vilnius University Vilnius Lithuania; ^12^ Epidemiology Department National Health Institute Doutor Ricardo Jorge Lisbon Portugal; ^13^ Croatian Institute of Public Health Zagreb Croatia; ^14^ Cantacuzino National Military‐Medical Institute for Research and Development Bucharest Romania; ^15^ University Hospital Brno Masaryk University Brno Czechia; ^16^ European Centre for Disease Prevention and Control Stockholm Sweden; ^17^ Infectious Diseases Department National Health Institute Doutor Ricardo Jorge Lisbon Portugal; ^18^ Sub‐Directorate General of Surveillance and Response to Public Health Emergencies, Public Health Agency of Catalonia Generalitat of Catalonia Barcelona Spain; ^19^ Department of Infectious Diseases Lithuanian University of Health Sciences Kaunas Lithuania; ^20^ Dr Victor Babes Clinical Hospital of Infectious and Tropical Diseases Bucharest Romania; ^21^ Instituto de Salud Pública de Navarra Navarra Spain; ^22^ Hospital Universitario de Navarra Navarra Spain; ^23^ Algemeen Ziekenhuis Sint‐Jan Brugge Belgium; ^24^ Centre Hospitalier de Wallonie Picarde Belgium; ^25^ Centre hospitalier Universitaire Saint‐Pierre Brussels Belgium; ^26^ CHU UCL Namur Université Catholique de Louvain Yvoir Belgium; ^27^ Grand Hôpital de Charleroi Belgium; ^28^ ZAS Hospital Antwerpen Belgium; ^29^ Jessa Ziekenhuis Hasselt Belgium; ^30^ Sciensano Belgium; ^31^ UZ Antwerpen Belgium; ^32^ UZ Brussels Belgium; ^33^ UZ Gent Belgium; ^34^ Department of Child & Adolescent Health Mater Dei Hospital Msida Malta; ^35^ Hungary; ^36^ HSE Health Protection Surveillance Centre Ireland; ^37^ Department for Infectious Disease Epidemiology RKI Germany; ^38^ Respiratory Diseases Clinic Heckeshorn Helios Klinikum Emil von Behring Berlin Berlin Lung Institut Germany; ^39^ Portugal; ^40^ Teaching Public Health Institute of Split‐Dalmatia County Croatia; ^41^ University Hospital Center Split Croatia; ^42^ Dr Victor Babes Clinical Hospital of Infectious and Tropical Diseases Bucharest Romania; ^43^ Sf Parascheva Clinical Hospital for Infectious Diseases Iasi Romania; ^44^ National Influenza Centre Cantacuzino National Military‐Medical Institute for Research and Development Romania

**Keywords:** case–control study, elderly, severe acute respiratory infections (SARI), test‐negative design, vaccine effectiveness

## Abstract

We estimated the effectiveness of the adapted monovalent XBB.1.5 COVID‐19 vaccines against PCR‐confirmed SARS‐CoV‐2 hospitalisation during the BA.2.86/JN.1 lineage‐predominant period using a multicentre test‐negative case–control study in Europe. We included older adults (≥ 65 years) hospitalised with severe acute respiratory infection from November 2023 to May 2024. Vaccine effectiveness was 46% at 14–59 days and 34% at 60–119 days, with no effect thereafter. The XBB.1.5 COVID‐19 vaccines conferred protection against BA.2.86 lineage hospitalisation in the first 4 months post‐vaccination.

## 1 Introduction

In the European Union/European Economic Area (EU/EEA), from 1 September 2023 to 15 April 2024, 99% of COVID‐19 vaccines administered as part of the 2023/24 vaccination campaigns were adapted monovalent XBB.1.5 COVID‐19 vaccines (XBB.1.5 vaccines), with Comirnaty accounting for 97% of them, when known [1].

The XBB.1.5‐like+F456L variant was circulating predominantly when the 2023 autumn vaccination campaigns started. In mid‐December, BA.2.86 SARS‐CoV‐2 lineage and sublineages, including JN.1, which were associated with potential immune escape [2, 3], started to dominate and effectiveness of the XBB.1.5 vaccines became of utmost interest [4].

We estimated the vaccine effectiveness (VE) of the XBB.1.5 vaccines against PCR‐confirmed SARS‐CoV‐2 hospitalisation in Europe during the BA.2.86/JN.1 lineage‐predominant period among older adults (≥ 65 years), by age group and time since vaccination (TSV).

## 2 VEBIS Hospital VE Network

**FIGURE 1:**
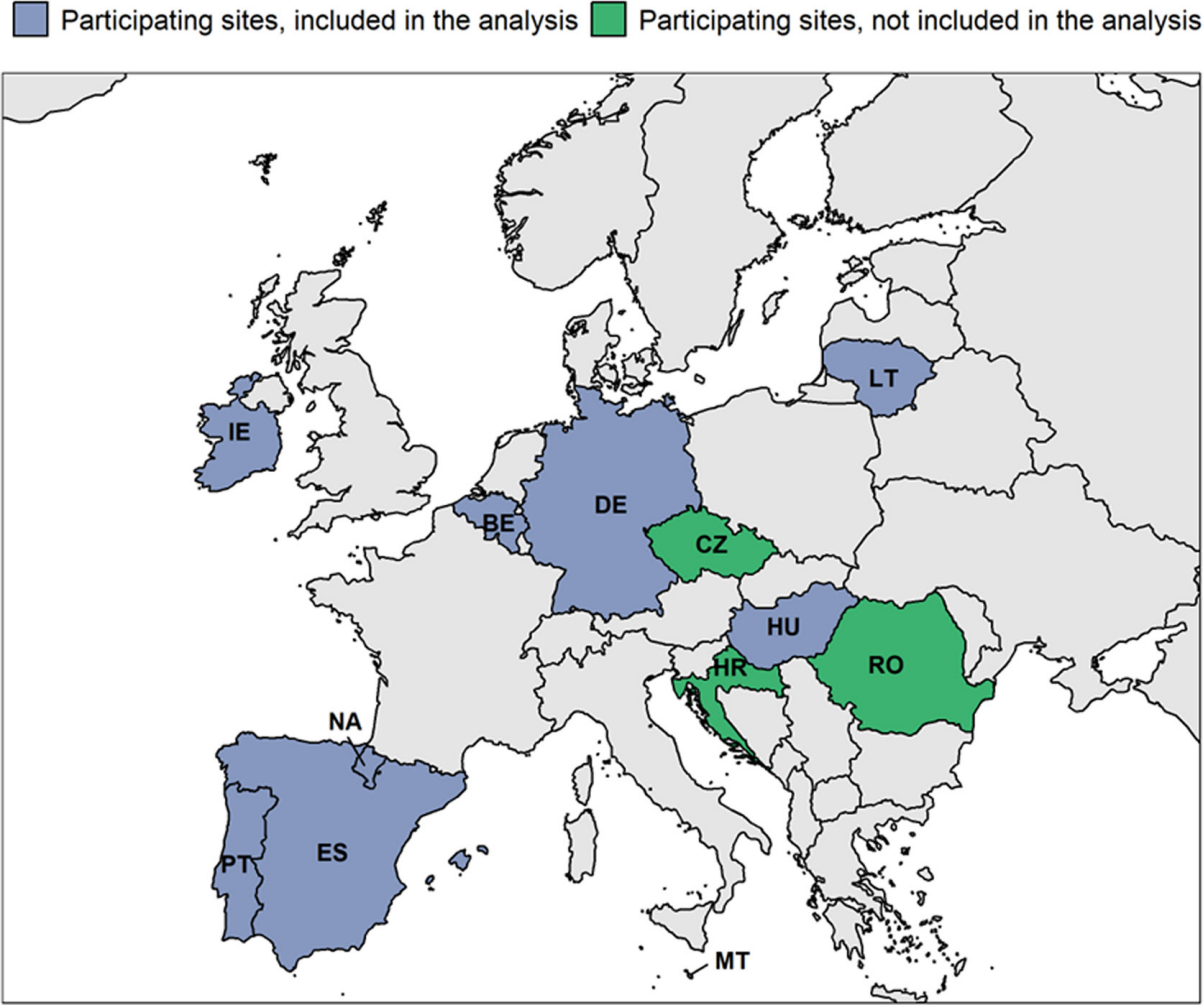
Countries and sites included in the VEBIS hospital network, Europe, 22 November 2023–18 May 2024. VEBIS: Vaccine Effectiveness, Burden and Impact Studies Twelve participating sites: Belgium (BE), Czechia (CZ), Germany (DE), Spain (ES), Croatia (HR), Hungary (HU), Ireland (IE), Lithuania (LT), Malta (MT), Navarre region, Spain (NA), Portugal (PT) and Romania (RO). Included in this analysis: BE, DE, ES, HU, IE, LT, MT, NA and PT.

This study, part of the Vaccine Effectiveness, Burden and Impact Studies (VEBIS), is a multicentre, test‐negative case–control study, including 77 hospitals in 11 participating European countries (Figure 1), following a common generic protocol [5].

Hospital teams recruit patients with severe acute respiratory infection (SARI) hospitalised for ≥ 24 h with at least one symptom among fever, cough, shortness of breath or sudden onset of anosmia, ageusia or dysgeusia [6]. Cases are SARI patients testing positive for SARS‐CoV‐2 by RT‐PCR within 48 h of admission or in the previous 14 days; controls are those testing negative.

We excluded patients with missing/erroneous information on key variables (sex, age, chronic conditions and dates of onset, swab and hospital admission) or vaccinated during the campaign with a COVID‐19 non‐XBB.1.5 vaccine (Figure S1). In Ireland and Portugal, we excluded patients with incomplete primary vaccination, as they were not eligible for XBB.1.5 vaccination during the 2023 autumn vaccination campaign (according to their vaccination guidelines).

We excluded sites with < 5 cases/controls or with no vaccinated SARI patients (Figure S1).

## 3 Definitions

The BA.2.86/JN.1 lineage‐predominant start was defined as the first week when ≥ 60% of all sequenced SARS‐CoV‐2 viral isolates were BA.2.86/JN.1 lineage within each country, based on data available at ECDC ERVISS GitHub (Table S2) [7]. The study period ended in the last week with data available for all sites.

We restricted analysis to patients aged ≥ 65 years. The study started 14 days after introduction of the XBB.1.5 vaccine, or after start of the BA.2.86/JN.1 predominant period, whichever was the latest in each study site (Tables S1 and S2).

We defined as vaccinated SARI patients with the last COVID‐19 vaccination dose received after the introduction of the XBB.1.5 vaccine in their country and as unvaccinated those who never received a COVID‐19 vaccine (with the exception of Portugal and Ireland), or with the last COVID‐19 vaccination dose ≥ 180 days prior to the start of the 2023/24 vaccination campaign. We excluded patients vaccinated 1–13 days before symptom onset.

We estimated the odds ratio (OR) of vaccination between cases and controls using logistic regression, adjusted for study site, date of symptom onset, sex, age and presence of a chronic condition. The best functional forms (categories, splines and linear terms) of continuous variables (age and onset date) were selected using Akaike's information criterion. VE was estimated as (1 − OR) × 100.

We present VE estimates by TSV in 60‐day bands and by age group (65–79, ≥ 80 years). To estimate the potential waning effect, we estimated the OR for each TSV band (60–119, ≥ 120 days), using 14–59 days as the reference.

We performed sensitivity analyses: (1) changing the criterion for ‘vaccinated’ from ≥ 14 to ≥ 7 days pre‐symptom onset; (2) excluding patients with the last dose 120 and 270 days instead of 180 days before the vaccination campaign starts; (3) excluding controls with known influenza or RSV co‐infections; (4) using an 80% threshold for BA.2.86/JN.1 predominant period.

## 4 SARI Patient Description and VE

We included 661 cases and 7386 controls aged ≥ 65 years, from 64 study sites, between 22 November 2023 to 18 May 2024, after exclusions (Figure S1). A total of 312 (47%) cases and 4333 (59%) controls were vaccinated (Figure S2). Median time between vaccination and symptom onset was 75 days for cases and 95 days for controls (Table 1).

**TABLE 1:** Characteristics of SARI patients by case and control status, VEBIS hospital study, Europe, 22 November 2023–18 May 2024 (*n* = 8047).

	SARS‐CoV‐2 cases (*n* = 661)		Test‐negative controls (*n* = 7386)	
SARI patient characteristic	*n*	%	*n*	%
Age (years)				
65–79	279	42	3658	50
≥ 80	382	58	3728	50
Median (IQR)	81 (75–87)		80 (73–86)	
Female	311	47	3629	49
Any chronic condition^a^	532	80	6101	83
Vaccination status at the time of symptom onset				
Vaccinated^b^	312	47	4333	59
Unvaccinated^c^	349	53	3053	41
Days since the last dose at the time of symptom onset^d^				
60‐day bands				
14–59 days	95	30	881	20
60–119 days	149	48	1900	44
≥ 120 days	68	22	1552	36
Median (IQR)	75 (55–109)		95 (65–140)	

Abbreviations: IQR: interquartile range; SARI: severe acute respiratory infection; VEBIS: Vaccine Effectiveness, Burden and Impact Studies.

^a^ Common chronic conditions: diabetes, heart disease, lung disease/asthma and immunodeficiency.

^b^ Received a COVID‐19 vaccine dose after the roll‐out of the XBB.1.5 vaccine in each country. For Portugal and Ireland, vaccinated patients were defined as those receiving at least their third COVID‐19 dose after the roll‐out of the XBB.1.5 vaccine or, if known, at least their second dose if the product of the primary series vaccination was Jcovden. Dates of the roll‐out of the XBB.1.5 vaccine are in Table S1.

^c^ Never‐vaccinated for COVID‐19 or with the last COVID‐19 vaccination dose received 180 days prior to the start of the 2023/24 vaccination campaign in each country. For Portugal and Ireland, the unvaccinated were individuals with at least primary series vaccination (only individuals previously vaccinated with at least primary series vaccination were eligible to receive an XBB.1.5 booster dose). Start dates of the 2023/24 vaccination campaign are in Table S1.

^d^ Restricted to those vaccinated with an XBB.1.5 vaccine during the 2023/24 vaccination campaign.

Among those ≥ 65 years old, VE overall was 45% (95% CI: 29; 58) in the first 14–59 days post‐vaccination, 34% (95% CI: 18; 47) for 60–119 days, and with no effect thereafter (Table 2).

**TABLE 2:** Vaccine effectiveness of adapted monovalent XBB.1.5 COVID‐19 vaccines against hospitalisation among SARI patients ≥ 65 years old during the BA.2.86/JN.1 variant‐predominant period, by TSV (60‐day bands) and by age group, VEBIS hospital study, Europe, 22 November 2023–18 May 2024 (*n* = 8047).

Age group	Vaccination status^a^/TSV (days)	SARI patient numbers		Days from the last dose to symptom onset^b^		VE^c^		Waning effect^d^	
		Cases	Controls	Median	IQR	%	95% CI	OR	95% CI
≥ 65 years^e^	Unvaccinated	349	3053	676	459–819	Ref.	Ref.	NA	NA
	14–59	95	881	45	34–53	45	29; 58	Ref.	Ref.
	60–119	149	1900	83	71–100	34	18; 47	1.2	0.9; 1.6
	120–235	68	1552	154	137–178	−10	−58; 24	2.0	1.3; 3.1
65–79 years	Unvaccinated	165	1738	738	470–827	Ref.	Ref.	NA	NA
	14–59	36	447	43	33–52	47	22; 65	Ref.	Ref.
	60–119	54	774	82	69–102	33	6; 53	1.3	0.8; 2.0
	120–234	24	699	154	137–180	−1	−78; 43	1.9	1; 3.8
≥ 80 years^e^	Unvaccinated	184	1315	545	450–804	Ref.	Ref.	NA	NA
	14–59	59	434	46	35–54	45	22; 61	Ref.	Ref.
	60–119	95	1126	84	72–100	32	9; 49	1.2	0.8; 1.8
	120–235	44	853	154	137–177	−18	−92; 27	2.1	1.2; 3.8

Abbreviations: CI: confidence interval; IQR: inter‐quartile range; OR: odds ratio; Ref.: reference category for logistic regression; SARI: severe acute respiratory infection; TSV: time since vaccination; VEBIS: Vaccine Effectiveness, Burden and Impact Studies.

^a^ Vaccinated: were those who received a COVID‐19 vaccine dose after the roll‐out of the XBB.1.5 vaccine in each country. For Portugal and Ireland, vaccinated patients were defined as those receiving at least their third COVID‐19 dose after the roll‐out of the XBB.1.5 vaccine or, if known, at least their second dose if the product of the primary series vaccination was Jcovden. Dates of the XBB.1.5 vaccine roll‐out are in Table S1; Unvaccinated: did not receive a vaccine during the campaign and were either never‐vaccinated for COVID‐19 or received their last COVID‐19 vaccination dose in the 180 days prior to the start of the vaccination campaign in their country. For Portugal and Ireland, the unvaccinated were individuals with at least primary series vaccination, received 180 days prior to the start of the vaccination campaign in their country (only individuals previously vaccinated with at least primary series vaccination were eligible to receive an XBB.1.5 booster dose). Start dates of the 2023/24 vaccination campaign are in Table S1.

^b^ Among patients who have received at least one COVID‐19 vaccine dose.

^c^ The OR of vaccination was estimated using a logistic regression model with site as a fixed effect and adjusted for date of symptom onset, sex, age and presence of any chronic condition (diabetes, heart disease, lung disease/asthma and immunodeficiency). The best functional forms of the continuous variables age and onset date (categories, splines and linear terms) were selected using the Akaike information criterion. Vaccine effectiveness is given by VE = (1 − OR) × 100.

^d^ The potential waning effect is estimated as the OR of vaccination between cases and controls for each later TSV band (60–119, ≥ 120 days), using the first band (14–59 days) as the reference group.

^e^ Maximum age included in the analysis: 105 years.

For those aged 65–79 years, VE was 47% (95% CI: 22; 65) in the first 14–59 days post‐vaccination and 33% (95% CI: 6; 53) at 60–119 days, with no effect thereafter (Table 2). For individuals aged ≥ 80 years, VE was 45% (95% CI: 22; 61) in the first 14–59 days post‐vaccination and 32% (95% CI: 9; 49) at 60–119 days, with no effect thereafter (Table 2).

The odds of COVID‐19–related hospitalisation increased with TSV, doubling at ≥ 120 days post‐vaccination compared with the first 14–59 days overall and across all age groups, with ORs of 2.0 (95% CI: 1.3; 3.1) for those aged ≥ 65 years, 1.9 (95% CI: 1; 3.8) for those aged 65–79 years and 2.1 (95% CI: 1.2; 3.8) for those aged ≥ 80 years.

In sensitivity analyses (1) and (2), the differences in VE estimates were ≤ 5% and ≤ 8% for (3). Larger differences were seen with some stratifications of sensitivity analysis (4), with VE lower by 19% absolute among those aged ≥ 80 years at 14–59 days post‐vaccination when a predominance threshold of 80% was used (Table S3).

## 5 Discussion

The adapted monovalent XBB.1.5 COVID‐19 vaccines conferred moderate protection among individuals aged ≥ 65 years during the BA.2.86/JN.1 lineage predominance, but VE waned over time from 45% (95% CI: 29; 58) at 14–59 days post‐vaccination, to 34% (95% CI: 18; 47) at 60–119 days, with no vaccine effect observed thereafter. Similar VE and potential waning effects were found across age groups.

Early XBB.1.5 VE results published from the VEBIS network (data from October 2023 to January 2024: a period of mostly XBB predominance) showed slightly higher estimates for those aged ≥ 80 years, at 14–29 days with 76% (95% CI: 53; 90) and at 30–59 days with 55% (95% CI: 34; 70) post‐vaccination [8], versus our 45% (95% CI: 29; 58) for 14–59 days post‐vaccination. Differences in point estimates cannot be explained only by different TSV, as the median TSV for the 30–59 days post‐vaccination group in the previously published analysis was similar to that for the 14–59 days post‐vaccination group in this analysis (47 vs. 45 days) [8]. Similarly, higher VE was found in the earlier analysis among those aged ≥ 65 years. For the 80% versus 60% predominance threshold within age groups, we found lower VE despite having the same median TSV. The lower VE may therefore be better explained by a higher immune escape of the BA.2.86/JN.1 lineages compared to XBB, rather than by TSV. As precision was low, and confidence intervals overlapped, random variation may also play a role.

Other European studies and two US studies found similar results; all suggested a lower VE against BA.2.86/JN.1 than XBB lineages [9, 10, 11, 12] albeit with some differences in study design and population [11, 12]. Results from other studies also suggested lower effectiveness against BA.2.86 than against XBB lineages [13, 14]. To the best of our knowledge, no other study has found no effect from the vaccine from 4 months post‐vaccination. Those reporting VE by TSV have either (1) shorter periods of observed TSV in their data [10, 11] and (2) used 90‐day TSV bands [9, 12], with low/moderate VE estimates at 90–179 days. Low sample size, depletion of susceptibles or low specificity of the outcome may also have contributed to this finding.

Although the cases included met the SARI case definition and were PCR‐positive for the SARS‐CoV‐2 virus, they might have been hospitalised for reasons unrelated to COVID‐19, which might underestimate VE [15]. Sensitivity analysis excluding patients with influenza or RSV co‐infections yielded similar results. In addition, analyses were conducted assuming that all vaccines administered after XBB.1.5 vaccine introduction in each country were XBB.1.5 vaccines, as vaccine brand/type was not systematically collected by all sites.

Due to the lack of information from all sites on the penultimate COVID‐19 dose received, we could not further minimise the risk of ineligibility, residual vaccination effect and potential VE estimate inflation by excluding patients vaccinated in the 2023/24 campaign who had received a previous COVID‐19 vaccine within 180 days prior to the start of the campaign (as we did for the unvaccinated). However, this only affected 7% of those vaccinated, of whom 3% were in the only country with a spring campaign.

Strengths of the study included its multi‐country component with a larger sample size enhancing population representativeness across Europe, providing a more generalisable pooled VE estimate. The use of a generic protocol mitigated potential sources of heterogeneity and increased internal validity.

Our results suggested that among older adults (≥ 65 years), protection of XBB.1.5 vaccines against SARS‐CoV‐2 BA.2.86/JN.1 hospitalisation in Europe was moderate up to 4 months post‐vaccination, with low to no effect thereafter.

## Author Contributions

**Liliana Antunes:** conceptualization, methodology, writing – original draft, writing – review and editing, visualization, validation, software, formal analysis, data curation, investigation. **Madelyn Rojas‐Castro:** investigation, methodology, writing – review and editing, formal analysis, software, validation, visualization, writing – original draft. **Marcos Lozano:** investigation, methodology, writing – review and editing, data curation, resources, supervision. **Iván Martínez‐Baz:** investigation, methodology, writing – review and editing, data curation, supervision, resources. **Isabel Leroux‐Roels:** investigation, methodology, writing – review and editing, data curation, supervision, resources. **Maria‐Louise Borg:** investigation, methodology, writing – review and editing, data curation, supervision, resources. **Beatrix Oroszi:** investigation, methodology, writing – review and editing, data curation, supervision, resources. **Margaret Fitzgerald:** investigation, methodology, writing – review and editing, data curation, supervision, resources. **Ralf Dürrwald:** investigation, methodology, writing – review and editing, data curation, supervision, resources. **Ligita Jancoriene:** investigation, methodology, writing – review and editing, data curation, supervision, resources. **Ausenda Machado:** investigation, methodology, writing – review and editing, data curation, supervision, resources. **Goranka Petrović:** investigation, methodology, writing – review and editing, data curation, supervision, resources. **Mihaela Lazar:** investigation, methodology, writing – review and editing, data curation, supervision, resources. **Lenka Součková:** investigation, methodology, writing – review and editing, data curation, supervision, resources. **Sabrina Bacci:** writing – review and editing, conceptualization, project administration, validation. **Jennifer Howard:** investigation, methodology, writing – review and editing, data curation, validation, visualization, software. **Nuno Verdasca:** investigation, methodology, writing – review and editing, data curation, supervision, resources. **Luca Basile:** investigation, methodology, writing – review and editing, data curation, supervision, resources. **Jesús Castilla:** investigation, methodology, writing – review and editing, data curation, supervision, resources. **Silke Ternest:** investigation, methodology, writing – review and editing, data curation, supervision, resources. **Aušra Džiugytė:** investigation, methodology, writing – review and editing, data curation, supervision, resources. **Gergő Túri:** investigation, methodology, writing – review and editing, data curation, supervision, resources. **Roisin Duffy:** investigation, methodology, writing – review and editing, data curation, supervision, resources. **Carolin Hackmann:** investigation, methodology, writing – review and editing, data curation, supervision, resources. **Monika Kuliese:** investigation, methodology, writing – review and editing, data curation, supervision, resources. **Verónica Gomez:** investigation, methodology, writing – review and editing, data curation, supervision, resources. **Zvjezdana Lovrić Makarić:** investigation, methodology, writing – review and editing, data curation, supervision, resources. **Alexandru Marin:** investigation, methodology, writing – review and editing, data curation, supervision, resources. **Petr Husa:** investigation, methodology, writing – review and editing, data curation, supervision, resources. **Nathalie Nicolay:** conceptualization, project administration, writing – review and editing, validation. **Angela M. C. Rose:** conceptualization, funding acquisition, supervision, project administration, methodology, writing – review and editing, investigation, writing – original draft, validation. **European Hospital Vaccine Effectiveness Group:** investigation, methodology, writing – review and editing, data curation, supervision, resources.

## Ethics Statement

The planning, conducting and reporting of the studies were in line with the Declaration of Helsinki. Official ethical approval was not required if studies were classified as being part of routine care/surveillance (Spain, Ireland, Malta); in Belgium and Germany, VE estimation is included in SARI surveillance. For Belgium, the study protocol was approved by the central Ethical Committee (CHU Saint‐Pierre [AK/12‐02‐11/4111] initially in 2011 and UZ VUB [B.U.N. 143201215671] from 2014 on) and each participating hospital's local ethical committees. The most recent amendment was approved on 27/9/2023 (reference 2012/310 Am6). The German SARI surveillance was approved by the Charité‐Universitätsmedizin Berlin Ethical Board (Reference EA2/218/19). Other study sites obtained local ethical approval from a national review board (Croatia: 3 July 2023 by the Ethics committee of the Croatian Institute of Public Health, Class 030‐02/23‐01/3; Hungary: approved in March 2021 by the National Scientific and Ethical Committee for the period 01 September 2021–01 September 2024 [IV/1885‐5/2021/EKU]; Lithuania: approved 11 May 2021 by Lithuanian Biomedical Research Ethics Committee, No. 6B‐21‐85; Navarra: PI2020/45; Portugal: approved 19 January 2021 by the Ethics Committee of Instituto Nacional de Saúde Doutor Ricardo Jorge, no registration number given; Romania: approved by the Ethics Committee of the Ministerul Apărării Naionale Institutul Naional de Cercetare pentru Dezvoltare Medico‐Militară, “Cantacuzino” for the period 2022–2023, No. CE199/2022).

## Consent

Written informed consent for participation and publication of data was obtained from all participants in accordance with ethical guidelines.

## Conflicts of Interest

Isabel Leroux‐Roels declares that her institution received funding from GSK, Janssen Vaccines, Moderna, MSD, Icosavax, Curevac, Moderna, Osivax, ICON Genetics and OSE Immunotherapeutics for the conduct of vaccine trials; from Janssen Vaccines and MSD for consulting services; and from Janssen Vaccines for participation on a data safety monitoring board and advisory board. All of these honoraria were paid to her institution.

Aukse Mickiene has received a grant for the Independent Investigator Initiated Research (Project Code/PO/Tracking Number WI236259; Grant ID#53233947); Pfizer R&D Investigator‐Initiated Research program (https://www.pfizer.com/science/collaboration/investigator‐initiated‐research) for the scientific project ‘A prospective study on the long‐term outcome and pathogenesis of tick‐borne encephalitis’, and a Grant from the European Society of Clinical Microbiology and Infectious Diseases (ESCMID) Study Group for Infectious Diseases of the Brain (ESGIB); sponsorship for participation in the international scientific conferences by MSD, Pfizer, Abbvie and Janssen; and payments for lectures in local scientific conferences and consultation fees from GSK, Sanofi, Pfizer, E‐visit.

Ligita Jancoriene has received honoraria fees for lectures from Pfizer, Viatris and Swixx Biopharma.

All other authors declare no conflicts of interest.

### Peer Review

The peer review history for this article is available at https://www.webofscience.com/api/gateway/wos/peer‐review/10.1111/irv.70081.

## Supporting information


**Figure S1.** Patient exclusion flowchart, VEBIS hospital study, November 2023–May 2024.
**Table S1.** Start date of the 2023/24 vaccination campaign by site, VEBIS hospital study, November 2023–May 2024.
**Table S2.** Start date and week number for the BA.2.86‐variant predominant period for 60% and 80% predominance thresholds, by site, VEBIS hospital study, November 2023–May 2024.
**Table S3.** Vaccine effectiveness of the adapted COVID‐19 XBB.1.5 vaccines against hospitalisation among individuals ≥ 65 years during the BA.2.86 lineage‐predominant period (80% threshold), by time since vaccination (60‐day bands) and by age group, VEBIS hospital study, Europe, 02 December 2023–20 May 2024 (*n* = 7820).
**Figure S2.** Number of cases and controls by week of symptoms onset, VEBIS hospital study, November 2023–May 2024.

## Data Availability

Data are available on request.
