# Supplementary material for: Effectiveness of the XBB.1.5 COVID‐19 Vaccines Against SARS‐CoV‐2 Hospitalisation Among Adults Aged ≥ 65 Years During the BA.2.86/JN.1 Predominant Period, VEBIS Hospital Study, Europe, November 2023 to May 2024
Source: Influenza Other Respir Viruses. 2025 Mar 9;19(3):e70081. doi: 10.1111/irv.70081 (PMC11890973; doi:10.1111/irv.70081)

## Supplementary Figure S1: Patient exclusion flowchart, VEBIS hospital study, Nov 2023–May 2024


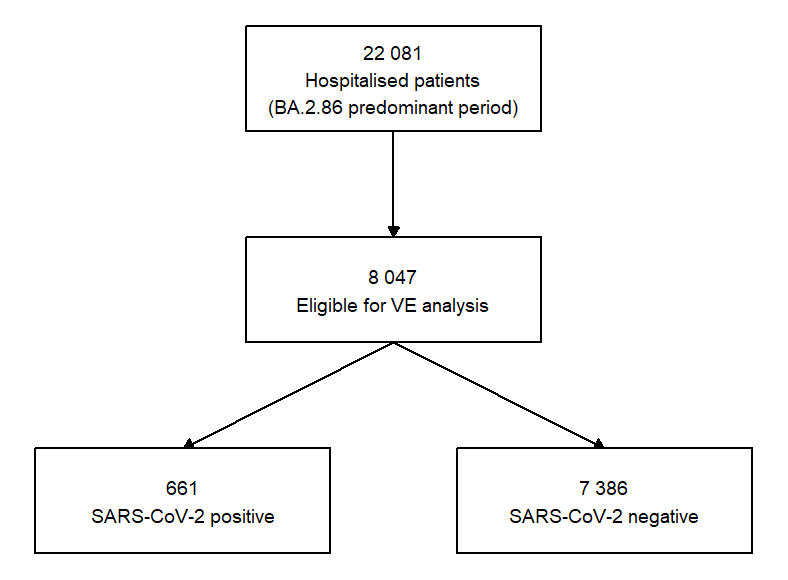


| **14 034 SARI patients excluded** |
| --- |
| - **4 245 according to per protocol exclusion criteria** |
| - - 839 were missing information on SARI case definition   - 1 057 did not meet the SARI case definition   - 2 349 had a missing RT-PCR test result/did not have an RT-PCR test |
| - **8 197 were in an ineligible population group** |
| - - 38 healthcare workers   - 865 residents in a long-term care facility   - 7 294 aged < 65 years |
| - **997 were missing key covariates for analysis** |
| - - 136 symptom onset date   - 4 age   - 8 sex   - 232 information on common chronic conditions   - 245 vaccination status   - 372 date of last received vaccine dose |
| - **185 were ineligible due to timing of symptom onset, swab and hospitalisation** |
| - - 176 swabbed > 48 hrs after hospitalisation   - 9 swabbed > 14 days before hospitalisation |
| - **136 with ineligible vaccination status** |
| - - 48 with last vaccine dose < 14 days before symptom onset   - 1 with contra indications for vaccine uptake   - 52 with last vaccine dose received within 180 days prior to the campaign   - 12 not eligible to be vaccinated in Portugal and Ireland   - 20 vaccinated with bivalent vaccine   - 1 vaccinated with vaccine brand other than Comirnaty, Spikevax or Nuvaxovid   - 2 received Comirnaty XBB.1.5 vaccine before 31 Aug 2023 |
| - **274 due to site restrictions** |
| - - 274 with no vaccinated patient (neither cases nor controls) |
| Records included are from 64 hospitals in nine sites: Belgium, Germany, Spain, Hungary, Ireland, Lithuania, Malta, Navarre region (Spain), and Portugal. |

## Supplementary Table S1: Start date of the 2023/24 vaccination campaign by site, VEBIS hospital study, Nov 2023–May 2024

| **Site** | **Date vaccination campaign started** | **XBB.1.5 vaccine introduction** |
| --- | --- | --- |
| Belgium | 15 Sep 2023 | 15 Sep 2023 |
| Czechia | 04 Aug 2023 | 04 Aug 2023 |
| Germany | 18 Sep 2023 | 18 Sep 2023 |
| Spain | 25 Sep 2023 | 25 Sep 2023 |
| Croatia | 18 Sep 2023 | 18 Sep 2023 |
| Hungary | 01 Oct 2023 | 01 Dec 2023 |
| Ireland | 02 Oct 2023 | 02 Oct 2023 |
| Lithuania | 05 Oct 2023 | 05 Oct 2023 |
| Malta | 09 Oct 2023 | 09 Oct 2023 |
| Portugal | 29 Sep 2023 | 29 Sep 2023 |
| Romania | 02 Oct 2023 | 17 Jan 2024 |

##

## Supplementary Table S2: Start date and week number for the BA.2.86-variant predominant period for 60% and 80% predominance thresholds ^a^, by site, VEBIS hospital study, Nov 2023–May 2024

| **Site** | **Start date (60% threshold)** | **Start date (80% threshold)** |
| --- | --- | --- |
| Belgium | 27 Nov 2023 | 11 Dec 2023 |
| Czechia | 11 Dec 2023 | 25 Dec 2023 |
| Germany | 04 Dec 2023 | 18 Dec 2023 |
| Spain | 04 Dec 2023 | 18 Dec 2023 |
| Croatia | 01 Jan 2024^b^ | 15 Jan 2024^b^ |
| Hungary | 25 Dec 2023^b^ | 15 Jan 2024^b^ |
| Ireland | 11 Dec 2023 | 25 Dec 2023 |
| Lithuania | 25 Dec 2023 | 01 Jan 2024 |
| Malta | 25 Dec 2023^b^ | 15 Jan 2024^b^ |
| Portugal | 27 Nov 2023^b^ | 18 Dec 2023^b^ |
| Romania | 01 Jan 2024^b^ | 1. an 2024^b^ |

^a^Data on SARS-COV-2 circulation available from ECDC ERVISS Github, extracted on 24 Sep 2024.

^b^Week start date calculated based on data from the country and neighbouring countries together: Croatia (neighbours = Hungary, Slovenia); Hungary (neighbours = Austria, Croatia, Romania, Slovenia, Slovakia); Malta (neighbours = Croatia, Greece, Italy); Portugal (neighbours = Spain); Romania (neighbours = Bulgaria, Hungary).

## Supplementary Table S3: Vaccine effectiveness of the adapted COVID-19 XBB.1.5 vaccines against hospitalisation among individuals ≥65 years during the BA.2.86 lineage-predominant period (80% threshold), by time since vaccination (60-day bands) and by age group, VEBIS hospital study, Europe, 02 Dec 2023–20 May 2024 (n = 7 820)

| Age group | Vaccination status^a^/ TSV | Number of SARI patients | | Days from last dose to symptom onset^b^ | | VE | | Waning effect | |
| --- | --- | --- | --- | --- | --- | --- | --- | --- | --- |
|  |  | Cases | Controls | Median | IQR | %^c^ | 95% CI | OR^d^ | 95% CI |
| ≥ 65 years^e^ | Unvaccinated | 267 | 2 782 | 676 | 459–819 | Ref. | Ref. | NA | NA |
|  | 14–59 days | 69 | 608 | 45 | 34–53 | 31 | 7;49 | Ref. | Ref. |
|  | 60–119 days | 147 | 1 880 | 83 | 71–100 | 30 | 13;45 | 1 | 0.7;1.4 |
|  | 120–235 days | 68 | 1 552 | 154 | 137–178 | -10 | -59;24 | 1.6 | 1;2.5 |
| 65–79 years | Unvaccinated | 131 | 1 582 | 732 | 470–827 | Ref. | Ref. | NA | NA |
|  | 14–59 days | 27 | 325 | 43 | 33–52 | 38 | 3;61 | Ref. | Ref. |
|  | 60–119 days | 54 | 765 | 82 | 69–102 | 31 | 3;52 | 1.1 | 0.7;1.8 |
|  | 120–234 days | 24 | 699 | 154 | 137–180 | -3 | -83;43 | 1.7 | 0.8;3.4 |
| ≥ 80 years^e^ | Unvaccinated | 136 | 1 200 | 545 | 450–804 | Ref. | Ref. | NA | NA |
|  | 14–59 days | 42 | 283 | 46 | 35–54 | 26 | -10;51 | Ref. | Ref. |
|  | 60–119 days | 93 | 1 115 | 84 | 72–100 | 28 | 3;47 | 1 | 0.6;1.5 |
|  | 120–234 days | 44 | 853 | 154 | 137–177 | -14 | -87;30 | 1.5 | 0.8;2.9 |

CI: confidence interval; OR: odds ratio; Ref.: reference category for logistic regression; SARI: severe acute respiratory infection; TSV: time since vaccination; VEBIS: Vaccine Effectiveness, Burden and Impact Studies.

aVaccinated: were those who received a COVID-19 vaccine dose after the roll-out of the XBB.1.5 vaccine in each country. For Portugal and Ireland, vaccinated patients were defined as those receiving at least their third COVID-19 dose after the roll-out of the XBB.1.5 vaccine or, if known, at least their second dose if the product of the primary series vaccination was Jcovden. Dates of the roll-out of the XBB.1.5 vaccine are in Supplementary Table S1; Unvaccinated: did not receive a vaccine during the campaign, and were either never-vaccinated for COVID-19, or received their last COVID-19 vaccination dose in the 180 days prior to the start of the vaccination campaign in their country. For Portugal and Ireland, the unvaccinated were individuals with at least primary series vaccination, received 180 days prior to the start of the vaccination campaign in their country (only individuals previously vaccinated with at least primary series vaccination were eligible to be receive an XBB.1.5 booster dose). Start dates of the 2023/24 vaccination campaign are in Supplementary Table S1.

^b^Median time since last COVID-19 vaccination dose in days and interquartile range among patients that have received at least one COVID-19 vaccine dose.

^c^The OR of vaccination is estimated using logistic regression model with site as a fixed effect and adjusted for date of symptom onset, sex, age and presence of any chronic condition (diabetes, heart disease, lung disease/asthma and immunodeficiency). The best functional forms of the continuous variables age and onset date (categories, splines, linear terms) were selected using the Akaike information criterion. Vaccine effectiveness is given by VE = (1-OR)%.

^d^The waning effect is estimated as the OR of vaccination between cases and controls for each time since vaccination band (60–119, ≥120 days) having as reference the first vaccination band (14–59 days).

^e^Maximum age included in the analysis: 105 years.

## Supplementary Figure S2: Number of cases and controls by week of symptoms onset, VEBIS hospital study, Nov 2023–May 2024


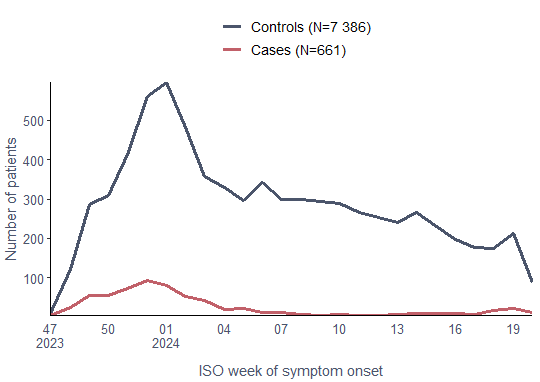

Supplement: Supplementary file 1 — Figure S1. Patient exclusion flowchart, VEBIS hospital study, November 2023–May 2024. Table S1. Start date of the 2023/24 vaccination campaign by site, VEBIS hospital study, November 2023–May 2024. Table S2. Start date and week number for the BA.2.86‐variant predominant period for 60% and 80% predominance thresholds, by site, VEBIS hospital study, November 2023–May 2024. Table S3. Vaccine effectiveness of the adapted COVID‐19 XBB.1.5 vaccines against hospitalisation among individuals ≥ 65 years during the BA.2.86 lineage‐predominant period (80% threshold), by time since vaccination (60‐day bands) and by age group, VEBIS hospital study, Europe, 02 December 2023–20 May 2024 (n = 7820). Figure S2. Number of cases and controls by week of symptoms onset, VEBIS hospital study, November 2023–May 2024. [file IRV-19-e70081-s001.docx]
